# A Global Fund to Fight Neglected Tropical Diseases: Is the G8 Hokkaido Toyako 2008 Summit Ready?

**DOI:** 10.1371/journal.pntd.0000220

**Published:** 2008-03-26

**Authors:** Peter J. Hotez, David H. Molyneux, Alan Fenwick, Lorenzo Savioli, Tsutomu Takeuchi

**Affiliations:** 1 Department of Microbiology, Immunology, and Tropical Medicine, The George Washington University and Sabin Vaccine Institute, Washington, D.C., United States of America; 2 Liverpool School of Tropical Medicine, Liverpool, United Kingdom; 3 Schistosomiasis Control Initiative, Imperial College London, United Kingdom; 4 Department of Neglected Tropical Diseases, World Health Organization, Geneva, Switzerland; 5 Department of Tropical Medicine and Parasitology, Keio University, Tokyo, Japan

The Global Fund to Fight AIDS, Tuberculosis and Malaria (“The Global Fund”) was established in 2002 as a partnership between governments, civil society, and the private sector to increase the global financing of three of the world's most devastating diseases in developing countries [1]. Under the executive directorship of Dr. Michel Kazatchkine, the Global Fund to date has attracted US$4.7 billion in financing through 2008, and in its first two rounds of grant making has committed an estimated US$1.5 billion to 93 developing countries [1]. By the spring of 2007, the Global Fund announced that it had provided antiretroviral treatments to more than 1 million HIV-infected individuals; it had treated 3 million tuberculosis patients with direct observed therapy; and distributed more than 30 million insecticide-treated bednets for malaria [2]. In its 5-year assessment, *The Lancet* declared that The Global Fund has “become a major force in global health, providing 20% of the donor funding for HIV/AIDS, 64% for malaria, and 70% for tuberculosis” [2].

Since 2005 we have advocated the need to establish a suitable financing mechanism to combat some of the most common and highest burden neglected tropical diseases (NTDs), namely ascariasis, trichuriasis, hookworm infection, schistosomiasis, lymphatic filariasis, onchocerciasis, and trachoma [3]–[10]. The political challenges to establish such a fund are formidable given the donor prioritization of HIV/AIDS, tuberculosis, and malaria. However, there are several reasons why a Global Fund–type mechanism would satisfy an urgent need to support NTD control and elimination. The NTDs represent the most common infections of the world's poorest people—“the bottom billion” [3]—and are arguably a major reason why the world's poorest people are unable to escape a vicious and downward spiral of destitution [3]–[10]. Despite their global public health and economic importance [3],[4], as well as the proven success of their control and even elimination in many settings [10], the NTDs have been overshadowed by “the big three diseases” targeted by The Global Fund. This is a tragic oversight because we are now in a unique position to control or eliminate some of the highest burden NTDs through integrated use of donated drugs developed initially by Pharma for human and veterinary infections in the industrialized world, such as ivermectin, azithromycin, albendazole, and mebendazole [9],[10]. Other NTD drugs, such as diethylcarbamazine (approximately US$0.01/treatment/year) and praziquantel (US$0.20/treatment/year), are available as extremely low-cost generics (although Merck KGaA recently announced the conclusion of a 10-year partnership with the World Health Organization to control schistosomiasis by donating 200 million tablets of praziquantel). Such drugs can also be used to treat the NTDs through mass drug administration, and since 1988, they have been administered by Ministries of Health with support of public–private partnerships, resulting in substantial reductions in the number of cases of these conditions where implementation has been achieved [3]. For example, the African Programme for Onchocerciasis Control reported that in 2006 48 million people were treated in 19 countries [11], the Global Programme to Eliminate Lymphatic Filariasis treated over 258 million people in 44 countries [12], the Schistosomiasis Control Initiative (SCI) has overseen the distribution of over 40 million treatments against schistosomiasis in six countries [13], and other partners have added 13 million schistosomiasis and 24 million deworming treatments to children globally [14]. These figures dwarf the reach of many other global health programs and have been demonstrably successful by alleviating morbidity and reducing transmission.

In order to scale up the delivery of the medicines for purposes of mass drug administration, the major public–private partnerships devoted to controlling NTDs have harmonized their activities through the creation of a Global Network for NTD Control [3]. By integrating the control of the most prevalent NTDs through preventive chemotherapy guidelines established by the World Health Organization, a package of NTD drugs could be administered at a small fraction of the cost for yearly antiretroviral treatments, direct observed therapy for tuberculosis, or even the costs of bednets and antimalaria chemotherapy [10].

The enormous adverse health and economic burdens of the NTDs, and the excellent results of (largely externally funded) NTD control initiatives over recent years, call for the establishment of more sustained global financing mechanisms such as those currently available for HIV/AIDS, tuberculosis, and malaria. It has been argued convincingly that bilateral donors may be inadvertently creating potential and actual distortions in developing country health care systems by an overriding focus on HIV/AIDS [15] and possibly other diseases, thereby crowding out resources for other diseases of the poor. The co-endemicity and the operational synergies between AIDS, tuberculosis, malaria, and the NTDs [5] argue strongly for including low-cost and cost-effective NTD control measures in the next round of The Global Fund [3], as well as large-scale bilateral initiatives from the United States government such as the President's Emergency Plan for AIDS Relief (PEPFAR) and the President's Malaria Initiative (PMI). At the First WHO Global Partners' Meeting on Neglected Tropical Diseases in April 2007, which brought the world's leading scientific and public health control experts on these conditions together with political leaders, health ministers from the endemic countries, as well as the major NGOs and public–private partnerships [16], His Excellency Mr. Blaise Compaore, the President of Burkina Faso, following prescient remarks by WHO Director General Margaret Chan, called for the establishment of a global NTD fund.

A significant fund to address NTDs could be achieved by expanding the mandate of the Global Fund, both by providing resources to be allocated for NTD control or elimination within the present system of bids from Country Coordinating Mechanisms (within the traditional calls for proposals from the Global Fund), and by adding expertise to the Global Fund Technical Review Committees. Given the marginal per capita annual costs of NTD interventions [3]–[10] compared with the other interventions, this may be prove to become a highly efficient approach.

Alternatively, donors could establish a special “NTD Fund” for the implementation of interventions to address these diseases specifically. The application mechanism could be similar to the Global Fund, which was designed to strengthen health systems and investigate opportunities for linkages with other programs. Such a fund or financing mechanism could be overseen by a board utilizing the strong technical resources of the World Health Organization. The World Health Organization has recently established an NTD Scientific and Technical Advisory Group, which is appointed by the Director-General, while the overall expertise of the World Health Organization has been re-organized to house it in a cluster together with the HIV/AIDS, tuberculosis, and malaria departments in order to maximize the opportunities for operational and technical synergies. The proposed central role for the World Health Organization together with the Global Network for NTD Control's alliance of NTD partnerships [3] in an NTD Fund suggests that models such as Polio Eradication or Stop TB should be explored. A characteristic of any fund must be transparency, accountability, and multilateral contributions. The NTD Fund would require these features as well as speed in response to country needs and drug distribution mechanisms, because an important element of NTD control is the timing of annual or semi-annual community-based drug distribution, particularly in relation to rainfall and geographic access [17].

We believe that the case for a much greater focus on the NTDs (the afflictions of the living majority of the poorest populations) by the global development community has already been made [3]–[10]. Establishment of such an NTD Fund is an ethical, equity, and human rights issue, and it is supported by independent evidence that the interventions are the most cost-effective available in public health [18], which would also improve education, strengthen health systems, and reduce poverty [3]–[10],[19],[20]. In the meantime, the initial seeds leading to a NTD Fund are being planted. Private organizations and individuals have provided significant support to the Global Network and their partners for NTD rapid impact packages in Africa, Asia, and the Americas [3], and President Bush recently announced that his administration will pledge $350 million for NTD control in Africa and elsewhere [21]. The US pledge, which would build upon an existing NTD Control Program sponsored by the US Agency for International Development [3], will likely require a new congressional appropriation and a commitment by a new administration taking office in January 2009.

An important next step would be to address global NTD control at the annual Group of Eight (G8) leaders summit. Despite inclusion of parasitic diseases in Prime Minister Tony Blair's Commission for Africa Report [3], there was no mention of the NTDs at the 2005 G8 summit held in Gleneagles, Scotland. In 2006, there was also no indication that NTDs were on the agenda at the G8 summit in St. Petersburg, Russia, or even at the preceding inaugural meeting of the G8 health ministers. NTDs again failed to make the agenda at the 2007 G8 summit in Germany. In 2008, Japan is hosting two major conferences, the Fourth Tokyo International Conference on African Development (TICAD IV) and the G8 Summit in Hokkaido Toyako [22]. Earlier, two global health–related initiatives on NTDs were initiated by the Government of Japan: the Hashimoto Initiative and Okinawa Infectious Diseases Initiative. These pioneering efforts have helped to draw the attention of the international community to the scourge of parasitic diseases, and are based partly on Japan's extensive past experiences in the control of these infections [23],[24]. Ultimately, nationwide community-based deworming efforts played a significant role in Japan's post-war development [23],[24], while similar national control programs had an important impact on reducing the burden of helminth infections in Korea and other Asian countries [25].

While gathered in Japan, the development community needs a robust discussion about the importance of the NTDs as global health, educational, and economic threats, and the opportunities for creating innovative financial mechanisms for low-cost and cost-effective integrated NTD control must take place at these important meetings. For too long the NTDs have been the forgotten diseases among forgotten people living in poverty. If politicians are serious about ending poverty, the needs of the bottom billion affected by these NTDs need to be addressed. The G8 leaders must work now to end this neglect and address the problems of the majority of the poorest. If they do not, it is unlikely that the Millennium Development Goals will be achieved. G8 leaders must recognize this reality. To believe that focusing on the big three is a panacea for improving global health is delusory [26]. As has been pointed out, there are 740 million Africans who are not infected with HIV/AIDS; they deserve a slice of the available interventions [27]. Economic and social development cannot take place when populations are burdened by chronic debilitating conditions that are so prevalent but can be so easily controlled or even eliminated as so many programs have proved. Using models similar to existing financial structures such as the Global Fund, Polio Eradication, or Stop TB, a comparatively modest amount of funds—in the range of $2 billion in total over 5 years—should be deposited and earmarked for treatment programs targeting the poorest populations in the poorest countries [28]. Countries could apply to this fund for support in parallel with applications to the donor pharmaceutical companies for drug donations. Such an approach would result in cures or morbidity reductions for those people currently infected, and it would impact transmission, or in some settings result in disease elimination. We believe that establishment of a global NTD financing mechanism represents one of the very most cost-effective and urgently needed approaches for sustainable poverty reduction.
